# Sinensetin suppresses influenza a virus-triggered inflammation through inhibition of NF-κB and MAPKs signalings

**DOI:** 10.1186/s12906-020-02918-3

**Published:** 2020-05-05

**Authors:** Jiashun Li, Xiang Jie, Xiaoli Liang, Ziyu Chen, Peifang Xie, Xiping Pan, Beixian Zhou, Jing Li

**Affiliations:** 1 Department of Respiratory, Affiliated Huadu Hospital, Southern Medical University (People′s Hospital of Huadu District), Huadu, Guangzhou, Guangdong 510800 P.R. China; 2 Huizhou third people’s hospital, Guangzhou Medical University, Guangdong, 516002 China; 3 State Key Laboratory of Respiratory Diseases, Guangzhou Institute of Respiratory Health, National Clinical Centre of Respiratory Disease, The First Affiliated Hospital, Guangzhou Medical University, Guangzhou, Guangdong 510120 P.R. China; 4 Institute of Respiratory Diseases, Department of Respiratory, The Affiliated Hospital of Guangdong Medical University, Zhanjiang, 524001 China; 5 Institute of Chinese Integrative Medicine, Guangzhou Medical University, Guangzhou, Guangdong 511436 P.R. China; 6 Department of Pharmacy, The People′s hospital of Gaozhou, Gaozhou, 525200 Guangdong China

**Keywords:** Sinensetin, Influenza a virus, Anti-inflammatory, NF-κB, P38 MAPK, ERK1/2 MAPK

## Abstract

**Background:**

Human respiratory system infected with influenza A virus (IAV) elicited a robust pro-inflammatory response that resulted in severe illness and even death. Currently, limited immunomodulator is available to counteract IAV-associated pneumonia in the clinic. Sinensetin, a polymethoxylated flavone with five methoxy groups, has been found to possess anti-agiogenesis, anti-inflammatory and anti-diabetic activities. However, the effects of sinensetin on IAV-triggered pro-inflammatory response remain unclear. In the present study, the anti-inflammatory effects and corresponding possible mechanism of sinensetin in IAV-infected A549 cells were subjected to investigations.

**Methods:**

The cytotoxic effects of sinensetin towards A549 cells was detected by MTT and LDH assays. The antiviral activity of sinensetin against influenza A virus was assayed in A549 cells with an engineered replication-competent influenza A virus carrying Gaussia luciferase reporter gene infection. The effect of sinensetin on influenza A virus-triggered inflammatory reaction was determined by qRT-PCR, Luminex assays, ELISA and Western blot.

**Results:**

Our results showed that sinensetin did not exhibit antiviral activity against A/PR/8/34 (H1N1). Meanwhile, sinensetin treatment significantly decreased IAV-induced expression of pro-inflammatory mediators at mRNA and protein levels, including IL-6, TNF-α, IP-10, IL-8 and MCP-1. Additionally, levels of cyclooxygenase (COX)-2 and the downstream product prostaglandin E_2_ (PGE_2_) up-regulated by IAV infection were dramatically suppressed by sinensetin. The mechanistic investigation revealed that sinensetin treatment suppressed the NF-κB transcriptional activity using the NF-κB reporter stable HEK293 cell line stimulated with TNF-α (20 ng/mL) or influenza H1N1 virus. Furthermore, sinensetin abrogated influenza H1N1 virus-induced activation of NF-κB, ERK1/2 MAPK and p38 MAPK signalings.

**Conclusion:**

Collectively, our results indicated that sinensetin has potential capacity to attenuate IAV-triggered pro-inflammatory response via inactivation of NF-κB, ERK1/2 MAPK and p38 MAPK signalings, which implied that sinensetin may be a promising candidate drug for influenza H1N1 virus infection therapeutics.

## Background

Annual seasonal influenza or occasional highly pathogenic avian influenza caused significant morbidity and mortality each year worldwide, especially among young children, the elderly, pregnant and immunocompromised individuals [1–4]. A modeling study estimated that influenza A virus infection causes approximately 300,000–650,000 seasonal influenza–associated respiratory deaths annually [5]. Due to viral genome reassorment or mutation, influenza infection was unmanageable with the current available medications including vaccination and anti-influenza agents (amantadine and oseltamivir) [6, 7].

Symptoms caused by viral infection varied from mild to severe, such as nasal obstruction, cough, headache, fever and even progression of acute respiratory distress syndrome (ARDS), which were closely related to respiratory tract inflammation [8, 9]. It has been suggested that elevation of pro-inflammatory mediators such as IL-6 and TNF-α in local or systemic accounted for the symptom formation in human with seasonal influenza virus infection [10–12]. Uncontrolled cytokine production (termed “cytokine storm”) in fatal cases of highly pathogenic H5N1 or H7N9 avian influenza virus infection has been found to be as the most common findings [13, 14]. The efficacy of corticosteroids treatment for influenza patients with pneumonia remained controversial. For example, in critically ill patients with severe influenza pneumonia, early combination oseltamivir and corticosteroids treatment seemed to confer a therapeutic benefit [15]. Conversely, retrospective studies revealed that corticosteroids treatment contributed to delay viral clearance and increase mortality in patients with H3N2 or 2009 H1N1 influenza virus infection [16, 17]. Therefore, corticosteroids were not recommended as routine drugs to treatment of patients with severe influenza pneumonia [18]. Based on these facts, development of novel immunomodulator or therapeutic approach for treatment of influenza pneumonia is an urgent clinical need.

Initiation of inflammatory reaction triggered by viral infection is dependent on several signaling cascades, including NF-κB and MAPKs (ERK1/2 and P38 MAPK). It’s well-recognized that the interaction between virus-derived pathogen-associated molecular patterns (PAMPs) and cellular specialized pattern recognition receptors (PPRs) leads to IKBα/NF-κB dissociation and thus released NF-κB translocates into nucleus, where it regulates the transcription of pro-inflammatory mediators, including TNF-α, IL-6, IL-8 and COX-2 [19]. Recent studies in vitro and in vivo revealed that massive induction of pro-inflammatory mediators in H5N1-infected patients was NF-κB-dependent [20, 21]. Furthermore, involvement of ERK1/2 and p38 MAPKs in the low or high pathogenic influenza-mediated dysregulation of pro-inflammatory cytokine expression has been reported [22, 23]. Therefore, these observations indicated that pharmacological inhibition of certain host cellular signaling associated with regulation of cytokine expression may act as a promising therapeutic strategy for the aberrant immune response induced by IAV.

Dietary intake of fruits rich in phytochemicals have been convincingly proved to have both nutritional and medicinal value, which provided potential beneficial effects for chemoprevention of various diseases, including influenza [24, 25]. Bioactive ingredient from edible fruits and vegetables have gained immense interest for development of novel agents to treat influenza diseases due to the broad safety window [26, 27]. Based on the available information in the literature, the natural polymethoxyflavone compound sinensetin has been found abundantly in citrus peel [28, 29]. Moreover, sinensetin was found to possess a number of pharmacological properties, including anti-inflammatory, anti-oxidative and anti-tumor [30–32]. Sinensetin has been reported to exert anti-inflammatory effects via inhibition of NF-κB signaling [33]. However, the effect of sinensetin on IAV infection remains to be elucidated.

In the current study, we speculated that plant-derived sinensetin may be considered as potential functional food ingredients that provided promising health benefits, and thus exert protective effects against influenza diseases. As proof of principle, we set out to examine the anti-inflammatory effects of sinensetin on IAV-induced pro-inflammatory response in A549 human lung epithelial cells as well as the mechanism underlying the anti-inflammatory effect of sinensetin on IAV infection.

## Methods

### Reagents

**Fig. 1:**
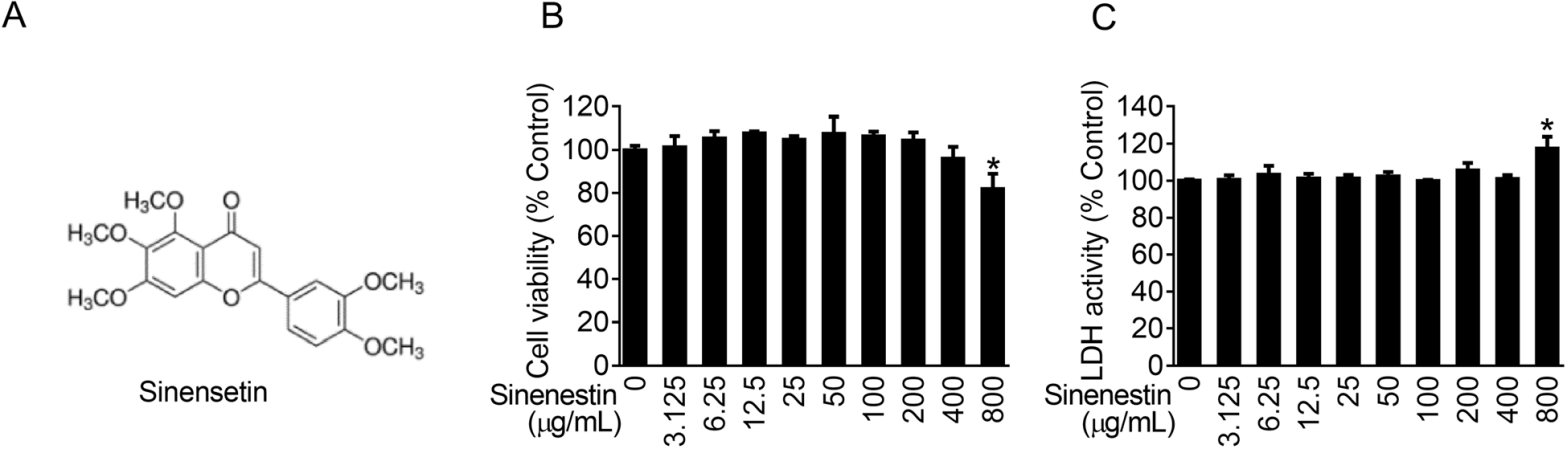
Effects of sinensetin on viability of A549 cells. **a** Chemical structure of sinensetin. **b** A549 cells (3 × 10^5^ cells/well) were treated with the indicated concentrations of sinensetin (0–800 μg/ml) for 48 h. Then, viability of A549 cells was determined by MTT assays. **c** Culture medium of A549 cells with the indicated concentrations of sinensetin (0–800 μg/ml) incubation for 48 h was collected and LDH activity was analyzed. Results are expressed as mean ± SEM. *P < 0.05 compared to untreated group

Sinensetin (Fig. 1a, Product No. S118322) was obtained from Aladdin Chemistry Co., Ltd. (Shanghai, China) (purity > 98%). Antibodies to P65, P38, ERK1/2, AKT, COX-2, GAPDH and phosphorylated form of P65, P38, ERK1/2 and AKT were from Cell Signaling Technology Inc. (Danvers, MA, USA). HRP-linked anti-rabbit secondary antibodies were purchased from EarthOx Life Science (Millbrae, CA, USA). BCA Protein Assay Kit for protein concentration quantification was purchased from Thermo Fisher Scientific Inc. (Walthman, MA, USA). The signal was detected using Enhanced chemiluminescence (ECL) reaction kit (Amersham Biosciences; Piscataway, NJ, USA). PrimeScript™ RT Reagent kit and Premix Ex Taq™ Reagent kit were obtained from Takara Bio, Inc. (Otsu, Japan).

### Cell lines, viruses and viral infections

Human type II alveolar epithelial cell line (A549) and Madin-Darby Canine Kidney (MDCK) cells were obtained from ATCC, and maintained in RPMI-1640 medium (Gibco) and DMEM medium (Gibco), respectively, both supplemented with penicillin-streptomycin (10 μL/mL) and 10% (v/v) heat-inactivated FBS in a humidified incubator with 5% CO_2_.

Influenza virus A/PR/8/34 (H1N1) used in present study was purchased from ATCC. The virus stocks were prepared in the allantoic cavities of embryonated chicken eggs and titrated on confluent MDCK cells in 96-well tissue culture plates.

For viral infection, A549 cells were inoculated with indicated multiplicities of infection (MOI) of viruses for 2 h, and then replaced with serum-free RPMI-1640 medium containing various concentrations of sinensetin.

### Cytotoxicity assay

The cytotoxicity of sinensetin on A549 cells was evaluated by MTT assay as described previously [34]. Briefly, A549 cells were cultivated into 96-well plates at a density of 5 × 10^4^ cells per well for 12 h incubation, allowing cells to adhere. Then, the cells were incubated with various concentrations of sinensetin (0–800 μg/mL). After 48 h incubation, the culture medium was discared and washed twice with phosphate-buffered saline (PBS). 100 μL of 0.5 mg/mL MTT solution (in DMEM medium) was added to each well and then incubated in dark for 4 h. Afterward, 150 μL of dimethyl sulfoxide (DMSO) was added to dissolve the formed formazan crystals. The absorbance at 570 nm was measured using a microplate reader (Biotek, Vermont, USA).

For lactated hydrogenase (LDH) assay, A549 cells (5 × 10^4^ cells/mL, 100 μL) were incubated with various concentrations of sinensetin (0–800 μg/mL). Following 24 h incubation, culture supernatants were harvested and the released LDH was measured using an LDH cytotoxicity assay kit (Beyotime, Nantong, China) according to the manufacturer’s protocol.

### Antiviral effect assay

A549 cells at a final density of 5 × 10^4^ cells per well were seeded into 96-well culture plates for 12 h incubation. Then, A549 cells were infected with 100TCID_50_ of IAV-Luc, an engineered replication-competent influenza A virus carrying Gaussia luciferase reporter gene as reported before [35]. Two hours later, the culutute supernatants containing viral inoculum were discarded and then cells were incubated with indicated concentration of sinensetin. After 24 h, cell culture supernatant was detected by adding with substrate of Coelenteron element and the signalings were assayed under the microplate Reader (Synergy HT, Bio-Tek, USA).

### Real-time RT-PCR

A549 cells seeded in 6-well plates were infected with influenza virus A/PR/8/34 (H1N1) (MOI = 0.1) for 2 h, and then inoculum was discarded and replaced with fresh serum free medium containing 0–120 μg/mL of sinensetin for 24 h. Total RNA was extracted from cells lysed in 1 mL of Trizol according to the manufacturers’ instructions. The PrimeScript™ RT Reagent kit was employed to yield first-strand complementary DNA (cDNA) from 1 μg of total RNA. Real-time quantitative PCR (qPCR) was performed to quantify the gene of interest with an Applied Biosystems 7500 system. Amplication conditions for qPCR were as follows: one cycle at 95 °C for 10 s, then followed by 40 cycles at 95 °C for 5 s and 60 °C for 40 s. Sequence of primers and probes are listed in supplementary Table 1. The 2^-△△CT^ method was employed to calculate relative target genes expression [36].

### Luminex

After IAV-infected cells in the presence or absence of sinensetin (0–120 μg/mL) incubation for 24 h, cell culture supernatants were harvested, centrifuged at 15000 rpm for 10 min at 4 °C to remove cellular debris, and stored at − 80 °C. Levels of cytokines in the supernatants were assayed by commercially available immunoassay kits (Millipore, Billerica, MA) according to the manufacturer’s protocol.

### Measurement of PGE_2_

The culture supernatants of IAV-infected cells in the presence or absence of sinensetin (0–120 μg/mL) were harvested at 24 h, and levels of prostaglandin E_2_ (PGE_2_) were assayed by ELISA Kit (Invitrogen) according to the manufacturer’s instructions.

### Western blotting

Monolayers of A549 cells were grown to 80–90% confluency in 6-well plates (Guangzhou Jet Bio-Filtration Co., Ltd, TCP-010-006) and infected with influenza virus A/PR/8/34 (H1N1) (MOI = 0.1) with different concentrations of sinensetin (0–120 μg/mL) treatment. After 24 h, cells were lysed in ice-cold radio-immunoprecipitation (RIPA) buffer (Beyotime, China) containing 1 mM phenylmethylsulphonylfluoride (PMSF) and protease inhibitors (Sigma-Aldrich; Merck, KGaA). Cellular lysates were centrifuged at 13,000 rpm at 4 °C for 15 min and then quantification of protein concentration was measured using a BCA protein assay kit (Pierce; Thermo Fisher Scientific, Inc.). Equal amounts of samples (20 μg) were electrophoresed by 10% SDS-PAGE and subsequently electro-transferred to PVDF membranes (0.2 μm, Bio-Rad, USA). After blocking with 5% non-fat milk in 1 x TBST (0.1% Tween − 20) for 1 h at room temperature, membranes were incubated overnight at 4 °C with indicated antibodies against COX-2, P-P65, P65, P-ERK1/2, ERK1/2, P-P38, P38, P-AKT, AKT and GADPH. Next, membranes were probed with horseradish peroxidase (HRP)-conjugated secondary antibodies and protein bands were visualized using an enhanced chemiluminescence (ECL) reaction kit. Relative intensities of bands were quantified using imageJ software vesion 1.44P (National Institutes of Health, Bethesda, MD, USA).

### NF-κB reporter assay

Monitoring of NF-κB activity is carried out based on reporter system as described previously [37]. Briefly, HEK293 (human embryonic kidney 293) cells, a stably-expressed NF-κB luciferase reporter and an internal control eGFP plasmid, were grown into 96-well plate for overnight to allow for attachment. Then, cells were stimulated with TNF-α (20 ng/mL) or infected with influenza virus A/PR/8/34 (H1N1) (MOI = 0.1) with or without sinensetin (0–120 μg/mL) treatment. After 24 h incubation, luciferase activity of cell lysates were measured using a luciferase assay kit (Promege). Luminescent signals were detected using a Synergy H1 Multi-Mode Reader (BioTek). The luciferase activities were normalized to parallel GFP intensities.

### Statistical analysis

All data were expressed as the mean ± standard error of the mean (SEM), and comparisons between different groups were performed by using one-way ANOVA analysis followed by Newman-Student-Keuls tests. *P* values less than 0.05 was considered statistically significant.

## Results

### Cytotoxicity effects of sinensetin on A549 cells

To avoid undesired biological effects caused by cytotoxic doses of sinensetin, we firstly carried out MTT assay to investigate the non-cytotoxic concentration range of sinensetin on A549 cells. As shown in Fig. 1b, sinensetin had no cytotoxicity on A549 cells at the concentration up to 400 μg/mL. Meanwhile, LDH assay showed that sinensetin did not lead to induce LDH release from A549 cells at the concentration up to 400 μg/mL (Fig. 1c), which suggested that sinensetin did not cause damage of plasma membrane. Therefore, we chose 120 μg/mL of sinensetin as the maximum dose for the further experiments.

### Effects of sinensetin on influenza a virus-induced expression of pro-inflammatory mediators

**Fig. 2:**
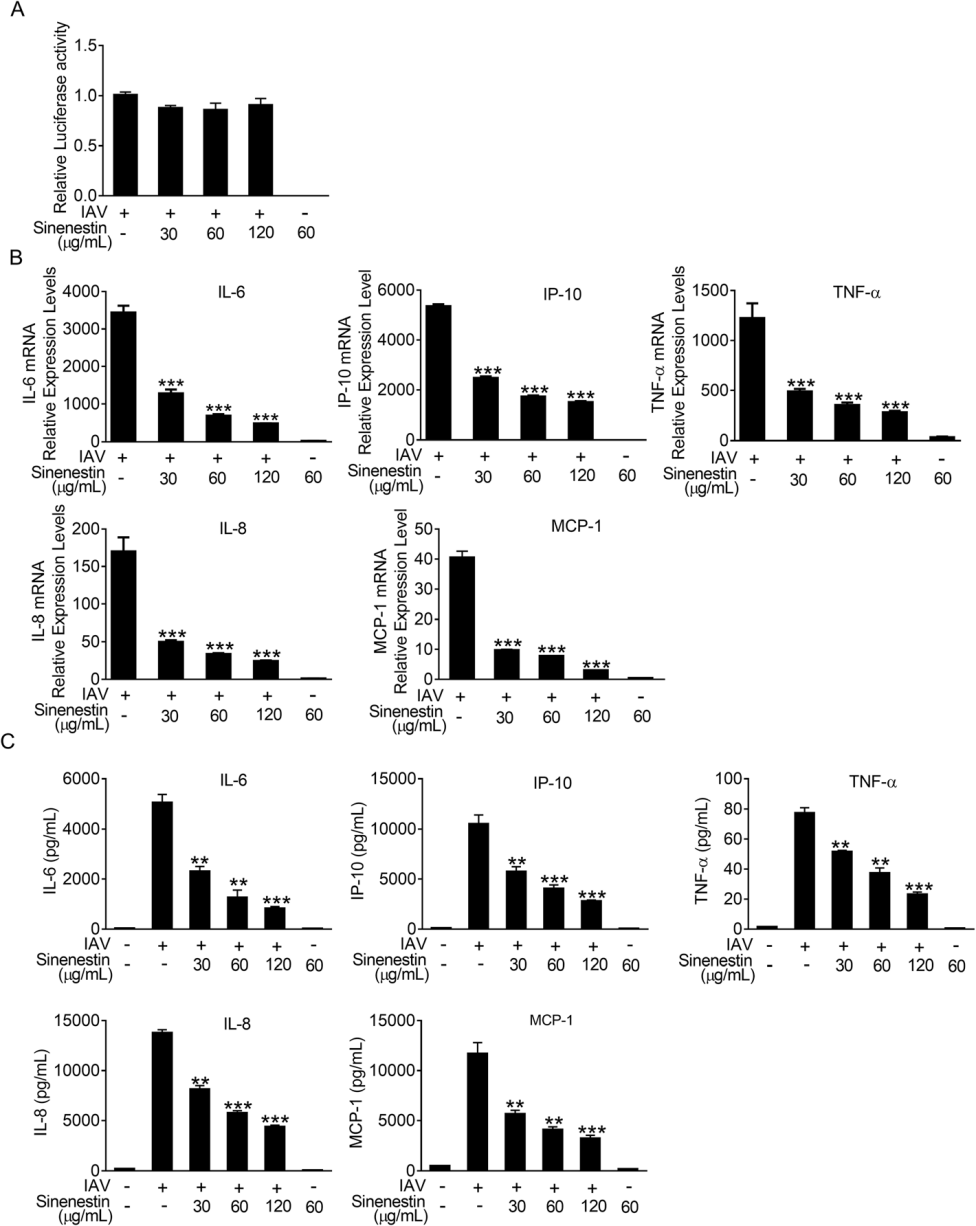
Sinensetin inhibited influenza A virus-induced expression of cytokines and chemokines in A549 cells. **a** Influenza A virus carrying Gaussia luciferase reporter was used for determination of the antiviral effects of sinensetin. **b**-**c** A549 cells were infected with A/PR/8/34 (H1N1) (MOI = 0.1) with or without sinensetin treatment for 24 h. The mRNA and protein levels of IL-6, IP-10, TNF-α, IL-8 and MCP-1 was determined by qRT-PCR (**b**) and Luminex (**c**). Data are expressed as mean ± SEM of three independent experiments. *P < 0.05, **P < 0.01, ***P < 0.001 compared to influenza virus-infected alone

Utilizing IAV carrying Gaussia luciferase reporter gene, we found that sinensetin did not exhibit antiviral activity against A/PR/8/34 (H1N1) (Fig. 2a). Besides, aberrant immune responses characterized by excessive pro-inflammatory mediator production or immunocytes recruitment could cause severe influenza illness [13, 38]. Therefore, we investigated effects of sinensetin on influenza A virus-mediated upregulation of pro-inflammatory mediators expression at mRNA and protein levels. At 24 h post-infection (p.i.), influenza virus A/PR/8/34 (H1N1) infection dramatically increased mRNA levels for pro-inflammatory mediators including IL-6, IP-10, TNF-α, IL-8 and MCP-1, while sinensetin treatment significantly reduced the expression of these cytokines and chemokines (Fig. 2b). Consistent with these results, influenza virus A/PR/8/34 (H1N1) -induced elevated secretion of IL-6, IP-10, TNF-α, IL-8 and MCP-1 in the culture supernatant was reversed by sinensetin treatment (Fig. 2c). These results indicated that sinensetin has the capacity to attenuate influenza virus-induced pro-inflammatory response.

### Effects of sinensetin on the production of influenza a virus-induced COX-2 and PGE_2_

**Fig. 3:**
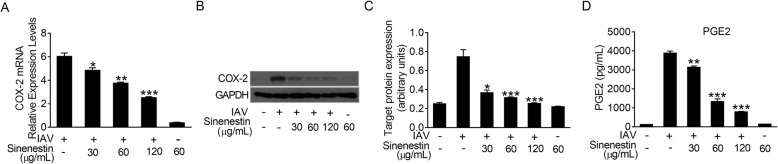
Sinensetin inhibited influenza A virus-induced expression of COX-2 and PGE_2_ in A549 cells. Influenza virus A/PR/8/34 (H1N1) (MOI = 0.1) infected A549 cells with or without indicated concentrations of sinensetin (30, 60, 120 μg/mL) treatment for 24 h. **a**-**b** COX-2 levels were detected by qRT-PCR (**a**) and immunoblot analysis (**b**), respectively. **c** COX-2 expression was quantified using the imageJ software. **d** PGE_2_ production in the culture supernatants was measured by ELISA. Data are expressed as mean ± SEM of three independent experiments. *P < 0.05, **P < 0.01, *** *P* < 0.001 compared to influenza virus-infected alone

Besides the cytokines and chemokines, lipid mediators such as PGE_2_ also played a critical pathogenic role during the influenza diseases [39]. Therefore, we carried out experiments to determine whether sinensetin treatment affected influenza virus-induced expression of lipid mediators. As expected, sinensetin treatment inhibited influenza A virus-induced both mRNA and protein expression levels of COX-2 in a dose-dependent manner (Fig. 3a, b). Parallel with these results, increased levels of cyclooxygenase (COX)-2 derived prostaglandin E_2_ (PGE_2_) by influenza virus infection were suppressed by sinensetin treatment (Fig. 3d). These data suggested that sinensetin was able to suppress influenza A virus-induced COX-2 expression and PGE_2_ production.

### Effects of sinensetin on the activation of NF-κB and MAPK signalings in influenza a virus-infected A549 cells

**Fig. 4:**
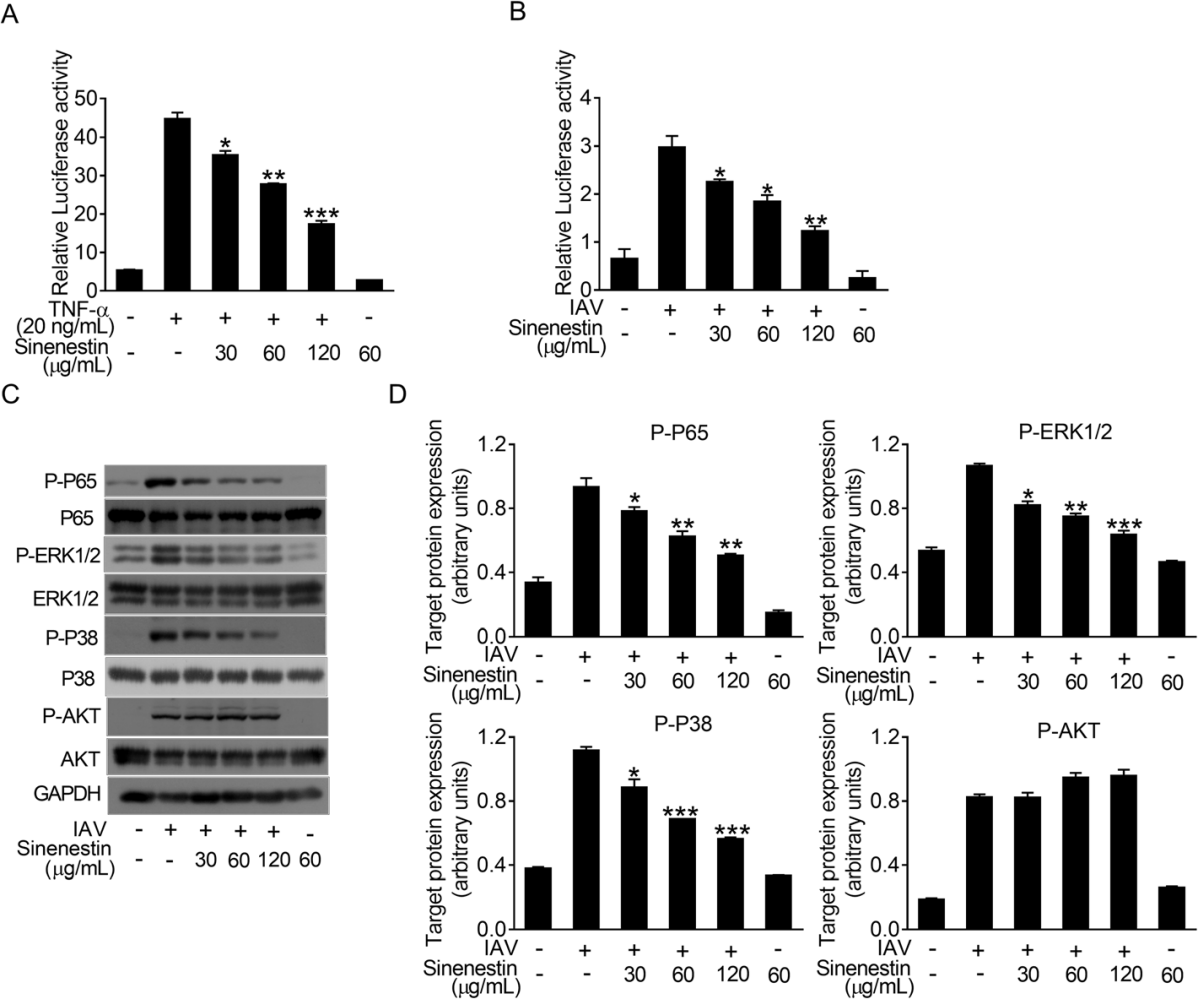
Sinensetin inhibited influenza A virus-induced activation of NF-κB and MAPK signalings. **a**-**b** NF-κB reporter stable cell line HEK293 cells were stimulated with TNF-α (20 ng/mL) (**a**) or influenza virus A/PR/8/34 (H1N1) (MOI = 0.1) (**b**) in the presence or in the absence of sinensetin (0, 30, 60,120 μg/mL). After 24 h incubation, cells were lysed for luciferase activity determination. **c** Immunoblot analysis of phosphorylated-P65, phosphorylated-ERK1/2, phosphorylated-P38 and phosphorylated-AKT in influenza virus A/PR/8/34 (H1N1)-infected A549 cells with or without sinensetin (0, 30, 60, 120 μg/mL) treatment. GAPDH was used as internal control. **d** Indicated phosphorylated protein band intensities were quantified by ImageJ software. Data are expressed as mean ± SEM of three independent experiments. *P < 0.05, **P < 0.01, ***P < 0.001 compared to influenza virus-infected alone

IAV-mediated host cellular signaling pathways activation have been believed to be responsible for excessive pro-inflammatory mediator production [40]. To reveal the mechanism underlying the inhibitory effects of sinensetin on influenza virus-induced pro-inflammatory reaction, we investigated cellular signaling transduction affected by sinensetin during viral infection. Firstly, the NF-κB reporter plasmid experiments showed that sinensetin treatment inhibited TNF-α or influenza virus A/PR/8/34 (H1N1) -stimulated activation of NF-κB (Fig. 4a, b). In accordance to that, immunoblot results confirmed that activation of NF-κB triggered by viral infection was inhibited by sinensetin treatment (Fig. 4c). Similarly, sinensetin treatment significantly decreased the activation of ERK1/2 and p38 MAPK signalings, but not AKT signaling. Collectively, these results demonstrated that sinensetin exerted an anti-inflammatory effect via blockade of NF-κB, ERK1/2 MAPK and p38 MAPK signalings.

## Discussion

Although antiviral medications such as oseltamivir were prescribed for prophylaxis and prevention of seasonal or pandemic influenza virus infection in clinic, patients with these drugs seem to be less effective due to delayed initiation of therapy [41, 42]. Accumulating evidence suggested that the severe IAV infection, for the most part, could be provoked or exacerbated by the extent of inflammation in the respiratory system [8]. Interestingly, delayed concomitant treatment of neuraminidase inhibitor and immunomodulator resulted in a therapeutic benefit effect in mice infected lethal H5N1 influenza virus [43], which highlighted that the development of effective immunomodulatory agents is essential to attenuate the severity of the viral infection.

In previous study, sinensetin was found to have the potential to reduce LPS-mediated release of pro-inflammatory cytokines via inactivation of NF-κB pathway [33]. But the effects of sinensetin on IAV-induced inflammation has not been reported. Our study demonstrated that sinensetin treatment significantly decreased IAV-triggered up-regulation of pro-inflammatory mediators, including IL-6, IP-10, TNF-α, IL-8, MCP-1, COX-2 and PGE_2_. Excessive production of pro-inflammatory mediators was considered as a hallmark of serious viral infectious diseases [44]. Increased IL-6 expression in patient with seasonal or pandemic influenza A H1N1 infection has been shown to be implicated in clinical symptom formation and illness severity [45]. Although TNF-α was documented for its role in antiviral immunity, it has pleiotropic properties that include stimulating other inflammatory mediator expression as well as disturbing the epithelial tight junction barrier, thus promoting development of ARDS [46–48]. In mice, studies demonstrated that deficiency in TNF-α signaling significantly improved highly virulent H5N1 virus-mediated excessive inflammation and high mortality [49]. Higher levels of chemokines, including IP-10, IL-8 and MCP-1, were believed to be associated with the acute respiratory distress syndrome (ARDS) progression and even fatal outcome in patients with H5N1 or H7N9 virus infection [14, 50]. Blockade of IP-10 and MCP-1 was proved to significantly lessen the disease severity of influenza virus infection [51, 52]. Expression of COX-2 and PGE_2_ has been suggested to play a role in enhancing virus-induced inflammation and impairment of host antiviral immunity [39, 53]. Based on the above evidences, the inhibitory effect on these IAV-induced inflammatory mediators exerted by sinensetin might act as a promising strategy for treatment of influenza infectious diseases with excessive inflammation.

Host intracellular signaling activated by viral infection, such as NF-κB and MAPKs pathways, has been reported to be involved in the aberrant pro-inflammatory response associated with poor clinical outcome [23, 54]. It has been shown that the induction of immune response-related genes in the highly pathogenic H5N1 virus infection was almost NF-κB-dependent [20]. It is believed that aberrant p38 MAPK activation is a critical driver for increased circulating levels of pro-inflammatory cytokine in patients with severe influenza [49]. Moreover, NF-κB and p38 MAPK signaling are involved in initiating expression of lipid mediators COX-2 and PGE_2_ [55, 56]. Therefore, targeting of these signalings may provide a potential therapeutic strategy for attenuating dysregulated inflammation in patients with severe influenza. Pharmacologic inhibition of NF-κB, ERK1/2 MAPK and p38 MAPK decreased an array of proinflammatory mediators, including IL-6, IL-8, TNF-α, MCP-1, COX-2 and PGE_2_, and thus improved the outcome of influenza diseases [23, 57–60]. Our results showed that the polymethoxylated flavone sinensetin decreased IAV-mediated activation of NF-κB, ERK1/2 MAPK and p38 MAPK signalings. Therefore, it can be speculated that sinensetin exerted anti-inflammatory effect on IAV-mediated inflammation that may be due to its inhibitory action on these intracellular signaling cascades.

## Conclusions

In conclusion, our data suggested that sinensetin attenuated IAV-induced pro-inflammatory cytokines and chemokines as well as lipid mediator, the mechanism of which is associated with the inhibition of IAV-mediated NF-κB, ERK1/2 MAPK and p38 MAPK signalings. Therefore, sinensetin could hold promise for the therapeutics of influenza diseases with excessive inflammation.

## Supplementary information

**Additional file 1: Table S1.** Primers and Probe Sequences for qRT-PCR.

## Data Availability

The datasets used and/o r analyzed during the current study is available from the corresponding author on reasonable request.

## Abbreviations

COX-2: Cyclooxygenase-2
IAV: Influenza A virus
IL-6: interleukin 6
IL-8: interleukin 8
IP-10: IFN-γ-inducible IP-10
LDH: Lactate dehydrogenase
LPS: Lipopolysaccharide
MAPK: Mitogen-activated protein kinase
MCP-1: Monocyte chemoattractant protein-1
MTT: 3-(4,5-dimethylthiazol-2-yl)-2,5-diphenyl tetrazolium bromide
NF-κB: Nuclear factor-κB
PGE_2_: Prostaglandin E2
TNF-α: Tumor necrosis factor alpha
