# Supplementary material for: Sinensetin suppresses influenza a virus-triggered inflammation through inhibition of NF-κB and MAPKs signalings
Source: BMC Complement Med Ther. 2020 May 5;20:135. doi: 10.1186/s12906-020-02918-3 (PMC7200050; doi:10.1186/s12906-020-02918-3)
Supplement: Supplementary file 1 — Additional file 1: Table S1. Primers and Probe Sequences for qRT-PCR. [file 12906_2020_2918_MOESM1_ESM.doc]

**Supplement Table 1**

Primers and Probe Sequences for qRT-PCR

| Gene | Primers and Probe | Sequence (5’→3’) |
| --- | --- | --- |
| IL-6 | Forward | CGGGAACGAAAGAGAAGCTCTA |
|  | Reverse | CGCTTGTGGAGAAGGAGTTCA |
|  | Probe | TCCCCTCCAGGAGCCCAGCT |
| IL-8 | Forward | TTGGCAGCCTTCCTGATTTC |
|  | Reverse | TATGCACTGACATCTAAGTTCTTTAGCA |
|  | Probe | CCTTGGCAAAACTGCACCTTCACACA |
| IP-10 | Forward | GAAATTATTCCTGCAAGCCAATTT |
|  | Reverse | TCACCCTTCTTTTTCAT-TGTAGCA |
|  | Probe | TCCACGTGTTGAGATCA |
| TNF-α | Forward | AACATCCAACCTTCCCAAACG |
|  | Reverse | GACCCTAAGCCCCCAATTCTC |
|  | Probe | CCCCCTCCTTCAGACACCCTCAACC |
| MCP-1 | Forward | CAAGCAGAAGTGGGTTCAGGAT |
|  | Reverse | AGTGAGTGTTCAAGTCTTCGGAGTT |
|  | Probe | CATGGACCACCTGGACAAGCAAACC |
| MIG | Forward | TCTTGCTGGTTCTGATTGGAGTG |
|  | Reverse | GATAGTCCCTTGGTTGGTGCTG |
|  | Probe | CAGGAACAGCGACCCTTTCTCACTACTGG |
| COX-2 | Forward | GAATCATTCACCAGGCAAATTG |
|  | Reverse | TTTCTGTACTGCGGGTGGAAC |
|  | Probe | TTCCTACCACCAGCAACCCTGCCA |
| GAPDH | Forward | GAAGGTGAAGGTCGGAGTC |
|  | Reverse | GAAGATGGTGATGGGATTTC |
|  | Probe | CAAGCTTCCCGTTCTCAGCC |
